# The Complex Quorum Sensing Circuitry of *Burkholderia thailandensis* Is Both Hierarchically and Homeostatically Organized

**DOI:** 10.1128/mBio.01861-17

**Published:** 2017-12-05

**Authors:** Servane Le Guillouzer, Marie-Christine Groleau, Eric Déziel

**Affiliations:** INRS-Institut Armand-Frappier, Laval, Québec, Canada; Georgia Institute of Technology

**Keywords:** *Burkholderia*, acyl-homoserine lactone, gene regulation, LuxR/LuxI, quorum sensing

## Abstract

The genome of the bacterium *Burkholderia thailandensis* encodes three complete LuxI/LuxR-type quorum sensing (QS) systems: BtaI1/BtaR1 (QS-1), BtaI2/BtaR2 (QS-2), and BtaI3/BtaR3 (QS-3). The LuxR-type transcriptional regulators BtaR1, BtaR2, and BtaR3 modulate the expression of target genes in association with various *N*-acyl-l-homoserine lactones (AHLs) as signaling molecules produced by the LuxI-type synthases BtaI1, BtaI2, and BtaI3. We have systematically dissected the complex QS circuitry of *B. thailandensis* strain E264. Direct quantification of *N*-octanoyl-homoserine lactone (C_8_-HSL), *N*-3-hydroxy-decanoyl-homoserine lactone (3OHC_10_-HSL), and *N*-3-hydroxy-octanoyl-homoserine lactone (3OHC_8_-HSL), the primary AHLs produced by this bacterium, was performed by liquid chromatography coupled to tandem mass spectrometry (LC-MS/MS) in the wild-type strain and in QS deletion mutants. This was compared to the transcription of *btaI1*, *btaI2*, and *btaI3* using chromosomal mini-CTX-*lux* transcriptional reporters. Furthermore, the levels of expression of *btaR1*, *btaR2*, and *btaR3* were monitored by quantitative reverse transcription-PCR (qRT-PCR). We observed that C_8_-HSL, 3OHC_10_-HSL, and 3OHC_8_-HSL are differentially produced over time during bacterial growth and correlate with the *btaI1*, *btaI2*, and *btaI3* gene expression profiles, revealing a successive activation of the corresponding QS systems. Moreover, the transcription of the *btaR1*, *btaR2*, and *btaR3* genes is modulated by cognate and noncognate AHLs, showing that their regulation depends on themselves and on other QS systems. We conclude that the three QS systems in *B. thailandensis* are interdependent, suggesting that they cooperate dynamically and function in a concerted manner in modulating the expression of QS target genes through a successive regulatory network.

## IMPORTANCE

Quorum sensing (QS) is a widespread bacterial communication system coordinating the expression of specific genes in a cell density-dependent manner and allowing bacteria to synchronize their activities and to function as multicellular communities. QS plays a crucial role in bacterial pathogenicity by regulating the expression of a wide spectrum of virulence/survival factors and is essential to environmental adaptation. The results presented here demonstrate that the multiple QS systems coexisting in the bacterium *Burkholderia thailandensis*, which is considered the avirulent version of the human pathogen *Burkholderia pseudomallei* and thus commonly used as an alternative study model, are hierarchically and homeostatically organized. We found these QS systems to be finely integrated into a complex regulatory network, including transcriptional and posttranscriptional interactions, and further incorporating growth stages and temporal expression. These results provide a unique, comprehensive illustration of a sophisticated QS network and will contribute to a better comprehension of the regulatory mechanisms that can be involved in the expression of QS-controlled genes, in particular those associated with the establishment of host-pathogen interactions and acclimatization to the environment.

## INTRODUCTION

Quorum sensing (QS) is a global regulatory mechanism of gene expression depending on bacterial density (1). Gram-negative bacteria typically possess homologues of the LuxI/LuxR system initially characterized in the bioluminescent marine bacterium *Vibrio fischeri* (2). The signaling molecules *N*-acyl-l-homoserine lactones (AHLs) produced by the LuxI-type synthases accumulate in the environment throughout bacterial growth, providing information on cell density. These AHLs activate the LuxR-type transcriptional regulators that modulate the expression of QS target genes, which usually contain a *lux* box sequence in their promoter region. These genes include a *luxI* homologue encoding a LuxI-type synthase generally located in close vicinity of a *luxR* homologue that codes for a LuxR-type transcriptional regulator, resulting in a typical self-inducing loop of AHLs (3).

Species belonging to the *Burkholderia* genus generally carry a unique AHL-based QS system referred as the CepI/CepR QS system (4). The CepI synthase is responsible for the production of *N*-octanoyl-homoserine lactone (C_8_-HSL), whereas the CepR transcriptional regulator modulates the expression of QS target genes in association with C_8_-HSL, including the *cepI* gene (4). Additionally, the *cepR* gene transcription can be autoregulated as well (5, 6). Multiple QS circuitries were also reported for several *Burkholderia* spp., such as the members of the *Bptm* group that consists of the nonpathogenic soil saprophyte *Burkholderia thailandensis* and the closely related pathogens *Burkholderia pseudomallei* and *Burkholderia mallei* responsible for melioidosis and glanders, respectively (7–9). QS was reported to be involved in the regulation of several virulence factors in *B. pseudomallei* and to be essential to its pathogenicity (10, 11). *B. thailandensis*, which is considered the avirulent version of *B. pseudomallei* (12), is commonly used as a surrogate model for the study of *B. pseudomallei*, which is considered a potential bioterrorism agent and whose manipulation is consequently restricted to biosafety level 3 (BSL3) laboratories. The members of the *Bptm* group contain homologous LuxI/LuxR QS systems that are involved in the biosynthesis of various AHLs (13–17). In *B. thailandensis*, the LuxI/LuxR QS systems are referred to as the BtaI1/BtaR1 (QS-1), BtaI2/BtaR2 (QS-2), and BtaI3/BtaR3 (QS-3) QS systems. The QS-1, QS-2, and QS-3 systems are also found in *B. pseudomallei*, whereas the QS-2 system is absent in *B. mallei* (18). These species also possess additional orphan *luxR* homologues, namely, *btaR4* (*malR*) and *btaR5* in *B. thailandensis* (7–9, 19).

The QS-1 system is composed of the *btaI1* and *btaR1* genes that code for the BtaI1 synthase and the BtaR1 transcriptional regulator, respectively. BtaI1 is responsible for the production of C_8_-HSL (13), and transcription of *btaI1* is positively modulated by BtaR1 (20). The BtaI2 synthase and the BtaR2 transcriptional regulator encoded by the *btaI2* and *btaR2* genes, respectively, constitute the QS-2 system. BtaR2 directly activates expression of *btaI2* involved in both *N*-3-hydroxy-decanoyl-homoserine lactone (3OHC_10_-HSL) and *N*-3-hydroxy-octanoyl-homoserine lactone (3OHC_8_-HSL) biosynthesis (16). The QS-3 system comprises the *btaI3* gene encoding the BtaI3 synthase that also catalyzes the synthesis of 3OHC_8_-HSL (13), as well as the BtaR3 transcriptional regulator, the product of the *btaR3* gene located next to *btaI3*.

The main goal of this study was to dissect the QS regulatory network of *B. thailandensis* E264 to reveal the interactions existing between the QS-1, QS-2, and QS-3 systems. Besides verifying previously proposed and established interactions, we uncovered several interconnections between the QS-1, QS-2, and QS-3 circuits, providing a comprehensive picture of the complex QS network in *B. thailandensis* E264. Ultimately, this study will contribute to a better appreciation of the QS regulatory mechanism of the expression of genes in *B. thailandensis*, and in particular those related to pathogenicity in *B. pseudomallei*.

## RESULTS

### The *B. thailandensis* QS-1, QS-2, and QS-3 systems are successively activated.

**FIG 1:**
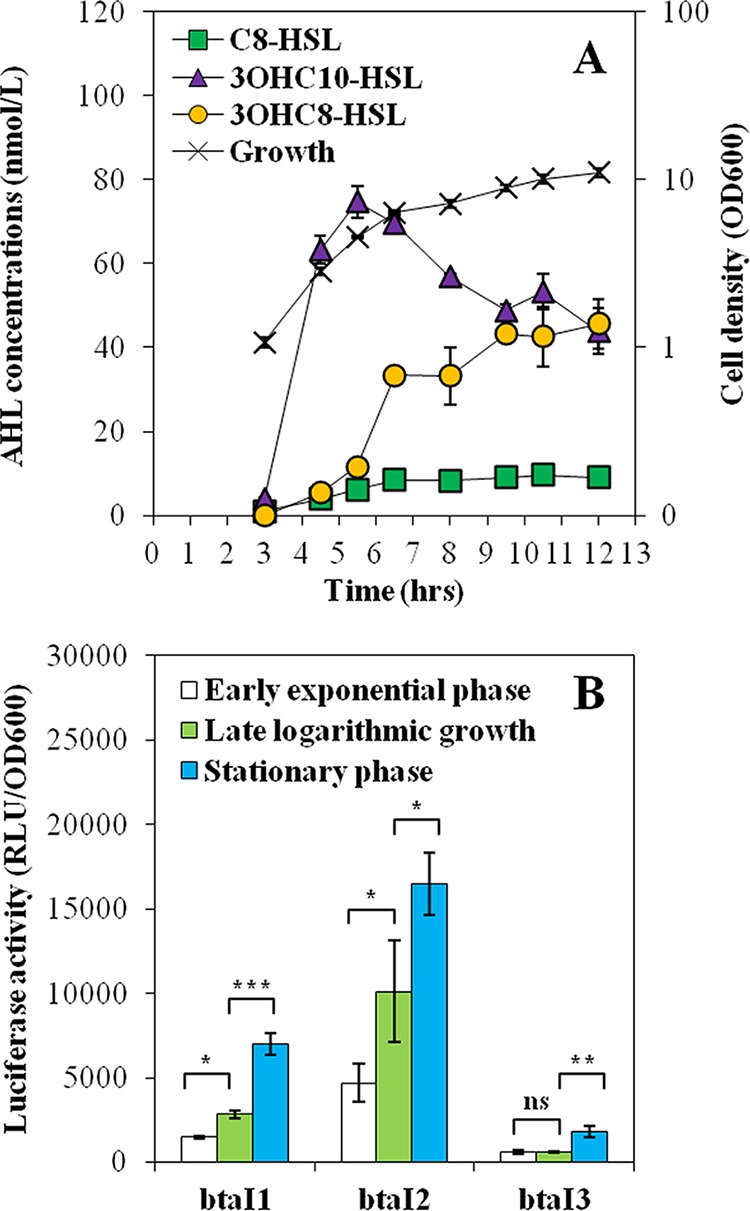
The QS-1, QS-2, and QS-3 systems are consecutively activated. (A) C_8_-HSL, 3OHC_10_-HSL, and 3OHC_8_-HSL concentrations were measured by LC-MS/MS throughout the different stages of bacterial growth in cultures of the wild-type E264 strain of *B. thailandensis*. The values are means ± standard deviations (error bars) for three replicates. (B) The luciferase activity of the chromosomal *btaI1*-*lux*, *btaI2*-*lux*, and *btaI3*-*lux* transcriptional fusions was monitored during the early exponential growth phase (OD_600_ ≈ 3.0), late logarithmic growth phase (OD_600_ ≈ 5.0), and stationary growth phase (OD_600_ ≈ 8.0). Luminescence is expressed in relative light units per optical density of the culture (RLU/OD_600_). Values that are significantly different are indicated by brackets and asterisks as follows: ***, *P* < 0.001; **, *P* < 0.01; *, *P* < 0.05. Values that are not significantly different (ns) are also indicated.

*B. thailandensis* E264 produces 3OHC_10_-HSL and to lesser extents, C_8_-HSL and 3OHC_8_-HSL (13, 16), but their levels at different stages throughout bacterial growth had never been investigated. Considering that nonsimultaneous production of AHLs in *B. pseudomallei* KHW was suggested (17), we hypothesized that these three AHLs are differentially produced over the growth phases of *B. thailandensis* E264. We thus determined the production profiles of C_8_-HSL, 3OHC_10_-HSL, and 3OHC_8_-HSL at various time points of the bacterial growth. Liquid chromatography coupled to tandem mass spectrometry (LC-MS/MS) was used to quantify the concentrations of these AHLs in wild-type *B. thailandensis* E264 cultures. We found that the amounts of 3OHC_10_-HSL increased rapidly through the early logarithmic growth phase (optical density at 600 nm [OD_600_] ≈ 3.0) and late exponential growth phase (OD_600_ ≈ 5.0) but decreased thereafter (Fig. 1A). Interestingly, 3OHC_8_-HSL concentrations kept increasing throughout bacterial growth to levels similar to the ones of 3OHC_10_-HSL (Fig. 1A). C_8_-HSL accumulated only during logarithmic growth and then remained stable in the stationary growth phase (OD_600_ ≈ 8.0; Fig. 1A).

To gain additional insights, biosynthesis of AHLs was correlated to the expression of the *btaI1*, *btaI2*, and *btaI3* genes. The activity of the chromosomal *btaI1*-*lux*, *btaI2*-*lux*, and *btaI3*-*lux* transcriptional reporters was measured during bacterial growth. In agreement with the AHL production profiles, activation of both *btaI1* and *btaI2* was observed from logarithmic growth (Fig. 1B), with *btaI2* expression starting earlier than for *btaI1* (data not shown), whereas *btaI3* was not activated until stationary phase was reached (Fig. 1B). Collectively, our results point toward a successive activation of the different QS systems in *B. thailandensis* E264 throughout the bacterial growth phases.

### The QS-1, QS-2, and QS-3 systems act in a coordinated way to finely modulate the synthesis of AHLs.

In order to verify whether the successive activation of the QS-1, QS-2, and QS-3 systems results from interactions between these QS circuits, we determined the kinetics of production of AHLs in cultures of the Δ*btaR1*, Δ*btaR2*, and Δ*btaR3* mutants compared to the wild-type E264 strain of *B. thailandensis* throughout the bacterial growth phases. We also measured expression of the AHL synthase-coding genes *btaI1*, *btaI2*, and *btaI3* in the same backgrounds harboring a chromosomal *btaI1*-*lux*, *btaI2*-*lux*, or *btaI3*-*lux* transcriptional fusion.

**FIG 2:**
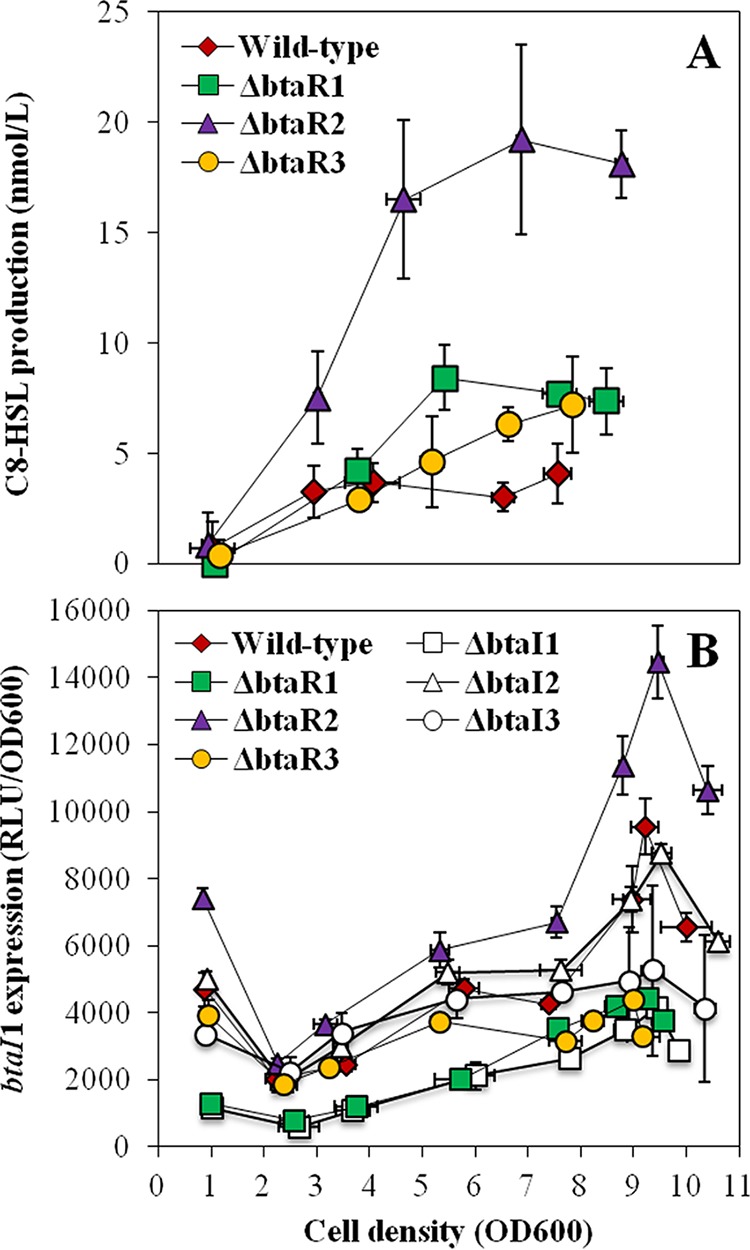
C_8_-HSL production and expression from the *btaI1* promoter in the wild-type and QS mutant strains of *B. thailandensis* E264. (A) The biosynthesis of C_8_-HSL was quantified using LC-MS/MS at various times during growth in cultures of the wild-type strain and of the Δ*btaR1*, Δ*btaR2*, and Δ*btaR3* mutant strains of *B. thailandensis* E264. The error bars represent the standard deviations of the averages for three replicates. (B) The luciferase activity of the chromosomal *btaI1*-*lux* transcriptional fusion was monitored in cultures of the wild-type strain and of the Δ*btaR1*, Δ*btaR2*, Δ*btaR3*, Δ*btaI1*, Δ*btaI2*, and Δ*btaI3* mutant strains of *B. thailandensis* E264. The luminescence is expressed in relative light units per optical density of the culture (RLU/OD_600_).

BtaI1 produces C_8_-HSL, and BtaR1 is considered the main regulator of *btaI1* expression (13). Therefore, we were surprised to see increased production of C_8_-HSL in the Δ*btaR1* mutant compared to the wild-type strain (Fig. 2A). This overproduction was principally detected after the end of the exponential phase. Nevertheless, transcription of the *btaI1* gene was lower in the Δ*btaR1* mutant throughout the different stages of bacterial growth, and it was almost zero in early logarithmic growth (Fig. 2B). Because of these results, it was important to confirm that *btaI1* expression is activated by BtaR1 in conjunction with C_8_-HSL. We monitored *btaI1* expression in response to exogenous addition of C_8_-HSL in the wild-type *B. thailandensis* strain E264 and its Δ*btaR1*, Δ*btaI1*, and Δ*btaI1* Δ*btaI2* Δ*btaI3* mutants. The *btaI1* gene exhibited comparable transcriptional profiles in the absence of BtaR1 or C_8_-HSL, supporting the idea that BtaR1/C_8_-HSL does indeed activate *btaI1* transcription (see Fig. S1 in the supplemental material). Accordingly, adding exogenous C_8_-HSL restored *btaI1* transcription in both the Δ*btaI1* and Δ*btaI1* Δ*btaI2* Δ*btaI3* mutants (Fig. S1). While expression of *btaI1* was induced in the wild-type strain culture supplemented with exogenous C_8_-HSL, no difference was noticed for the Δ*btaR1* mutant, confirming that activation of *btaI1* by this AHL involves BtaR1 (Fig. S1).

10.1128/mBio.01861-17.1 FIG S1  *btaI1* activation requires BtaR1 and C_8_-HSL. The luciferase activity of the chromosomal *btaI1*-*lux* transcriptional fusion was monitored during the exponential growth phase in cultures of the *B. thailandensis* E264 wild-type strain and the Δ*btaR1*, Δ*btaI1*, and Δ*btaI1* Δ*btaI2* Δ*btaI3* mutant strains. Cultures were supplemented with 10 µM C_8_-HSL. Acetonitrile only was added to the controls. The values represent the means for three replicates. The luminescence is expressed in relative light units per optical density of the culture (RLU/OD_600_). Download FIG S1, PDF file, 0.04 MB. Copyright © 2017 Le Guillouzer et al. 2017 Le Guillouzer et al. This content is distributed under the terms of the Creative Commons Attribution 4.0 International license.

To determine whether the QS-1 system is also under BtaR2 and BtaR3 control, we investigated the effects of these transcriptional regulators on both the production of C_8_-HSL and expression of *btaI1*. Interestingly, C_8_-HSL concentrations were also increased in the Δ*btaR*2 mutant, with a matching upregulation of *btaI1* expression during logarithmic growth (Fig. 2), revealing that BtaR2 might repress the production of C_8_-HSL by modulating the transcription of *btaI1*. While C_8_-HSL was also overproduced in the absence of BtaR3 during stationary phase (Fig. 2A), *btaI1* transcription was downregulated in the Δ*btaR*3 mutant (Fig. 2B), suggesting that the negative impact of BtaR3 on C_8_-HSL biosynthesis is indirect and does not result from *btaI1* regulation. Altogether, these data indicate that while BtaR1 constitutes the main regulator of the QS-1 system, C_8_-HSL biosynthesis is also directly and indirectly dependent on both BtaR2 and BtaR3, respectively.

**FIG 3:**
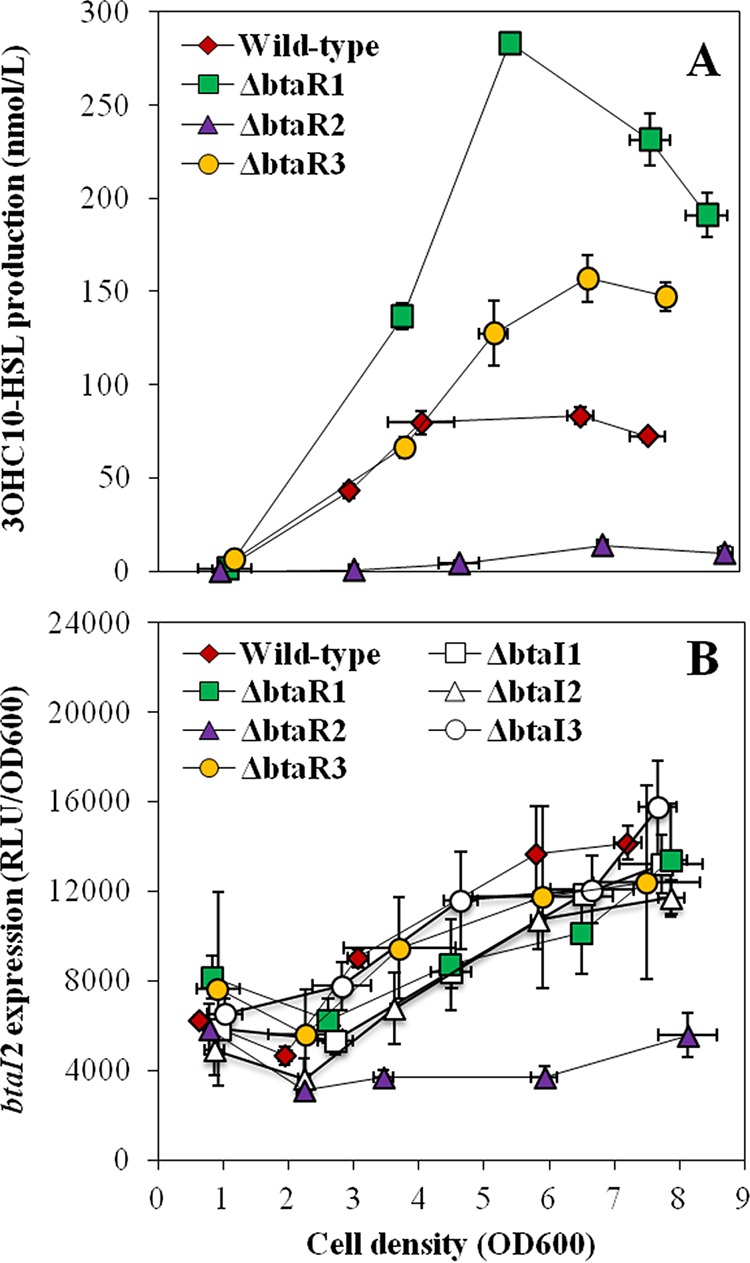
3OHC_10_-HSL production and expression from the *btaI2* promoter in the wild-type strain and QS mutant strains of *B. thailandensis* E264. (A) The biosynthesis of 3OHC_10_-HSL was quantified using LC-MS/MS at various times during growth in cultures of the wild-type and Δ*btaR*1, Δ*btaR*2, and Δ*btaR*3 mutant strains of *B. thailandensis* E264. The error bars represent the standard deviations of the averages for three replicates. (B) The luciferase activity of the chromosomal *btaI2*-*lux* transcriptional fusion was monitored in cultures of the wild-type and Δ*btaR1*, Δ*btaR2*, Δ*btaR3*, Δ*btaI1*, Δ*btaI2*, and Δ*btaI3* mutant strains of *B. thailandensis* E264. The luminescence is expressed in relative light units per optical density of the culture (RLU/OD_600_).

3OHC_10_-HSL is produced by the BtaI2 synthase (16). While BtaR2 directly activates *btaI2* expression in response to 3OHC_10_-HSL and 3OHC_8_-HSL, the latter being also produced by BtaI2 (16), the direct impact of BtaR2 on the production of these two AHLs is still untested. We observed that both 3OHC_10_-HSL biosynthesis and *btaI2* expression were almost completely abolished in the Δ*btaR2* mutant, confirming that BtaR2 is their main regulator (Fig. 3). Despite the absence of BtaR2, we detected a slight, but consistent and highly reproducible, production of 3OHC_10_-HSL during stationary phase (Fig. 3A). Accordingly, transcription of *btaI2* was also slightly augmented later (Fig. 3B). Thus, 3OHC_10_-HSL biosynthesis and *btaI2* expression might not be exclusively under BtaR2 control.

To determine whether BtaR1 and BtaR3 also intervene in the regulation of 3OHC_10_-HSL production and *btaI2* transcription, their effects on the QS-2 system were investigated. Interestingly, 3OHC_10_-HSL concentrations were strongly increased in the Δ*btaR1* mutant compared to the wild-type strain from the beginning of logarithmic growth (Fig. 3A). The levels of 3OHC_10_-HSL were also increased in the Δ*btaR3* mutant background, but this was observed only after the end of the exponential phase (Fig. 3A). However, in both cases, no impact on *btaI2* transcription was noticed despite an increase in the amounts of 3OHC_10_-HSL (Fig. 3B). Collectively, these observations indicate that although BtaR1 and BtaR3 influence the biosynthesis of 3OHC_10_-HSL, the effects of these transcriptional regulators on the QS-2 system are indirect.

**FIG 4:**
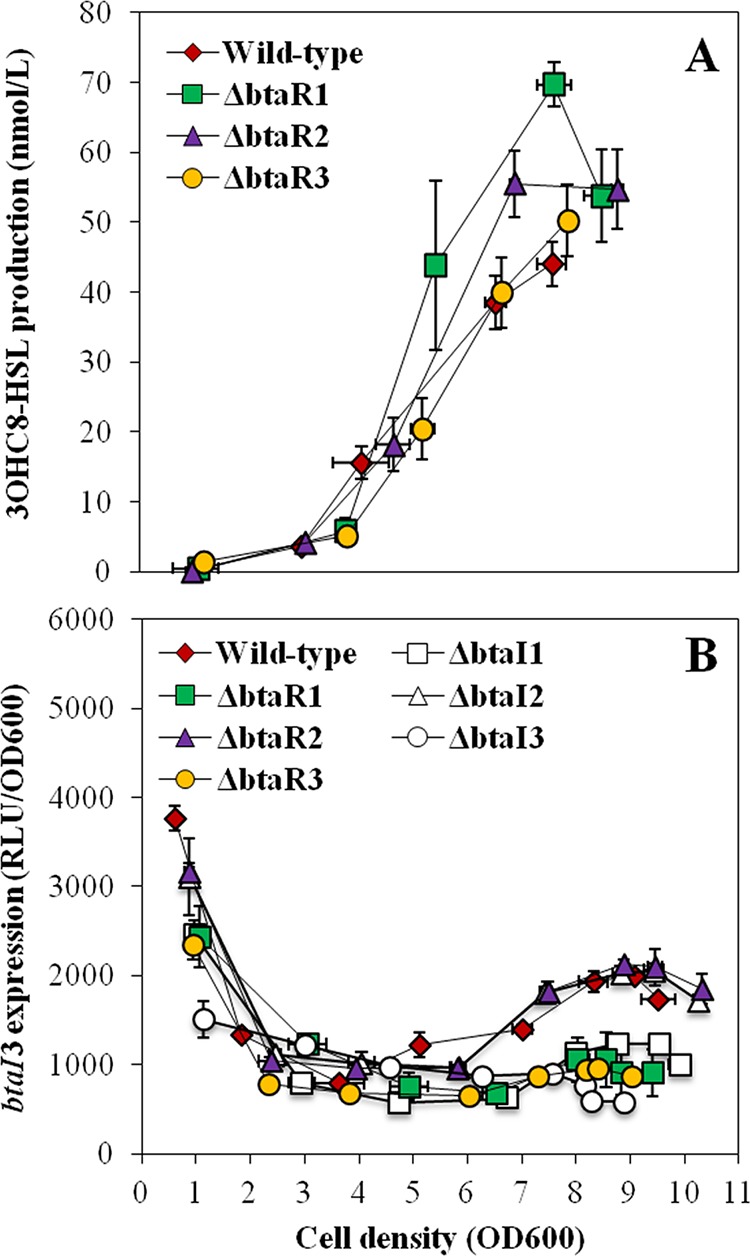
3OHC_8_-HSL production and expression from the *btaI3* promoter in the wild-type and QS mutant strains of *B. thailandensis* E264. (A) The biosynthesis of 3OHC_8_-HSL was quantified using LC-MS/MS at various times during growth in cultures of the wild-type and Δ*btaR1*, Δ*btaR2*, and Δ*btaR3* mutant strains of *B. thailandensis* E264. The error bars represent the standard deviations of the averages for three replicates. (B) The luciferase activity of the chromosomal *btaI3*-*lux* transcriptional fusion was monitored in cultures of the wild-type and Δ*btaR1*, Δ*btaR2*, Δ*btaR3*, Δ*btaI1*, Δ*btaI2*, and Δ*btaI3* mutant strains of *B. thailandensis* E264. The luminescence is expressed in relative light units per optical density of the culture (RLU/OD_600_).

BtaI3 is mainly responsible for 3OHC_8_-HSL biosynthesis (13). While no discernible difference in 3OHC_8_-HSL concentrations was detected in cultures of the Δ*btaR3* mutant compared to cultures of the wild-type strain (Fig. 4A), the levels of *btaI3* transcription were decreased (Fig. 4B). To confirm whether transcription of *btaI3* is dependent on BtaR3 and on 3OHC_8_-HSL, *btaI3* expression was measured in the wild-type strain and in the Δ*btaR3*, Δ*btaI3*, and Δ*btaI1* Δ*btaI2* Δ*btaI3* mutants supplemented with exogenous 3OHC_8_-HSL or not supplemented with 3OHC_8_-HSL. We found that *btaI3* was similarly downregulated in the Δ*btaR*3 and Δ*btaI3* mutant backgrounds, suggesting that BtaR3 activates *btaI3* in response to 3OHC_8_-HSL (Fig. S2). Accordingly, *btaI3* transcription was not affected by the addition of 3OHC_8_-HSL in the Δ*btaR*3 mutant, but it was increased in the wild-type strain culture under the same conditions, revealing that activation of *btaI3* by this AHL is linked to BtaR3 (Fig. S2). Unexpectedly, adding exogenous 3OHC_8_-HSL to the culture of the Δ*btaI3* mutant did not restore *btaI3* transcription to wild-type levels (Fig. S2). However, we observed that expression of *btaI3* was restored in the AHL-defective Δ*btaI1* Δ*btaI2* Δ*btaI3* mutant supplemented with 3OHC_8_-HSL, confirming the involvement of this AHL in the activation of *btaI3* (Fig. S2). Taken together, these data confirm that *btaI3* is activated by BtaR3/3OHC_8_-HSL and suggest that expression of this gene is controlled by additional AHLs and/or alternative LuxR-type transcriptional regulators.

10.1128/mBio.01861-17.2 FIG S2  *btaI3* is activated by BtaR3 and 3OHC_8_-HSL. The luciferase activity of the chromosomal *btaI3*-*lux* transcriptional fusion was measured during stationary phase in cultures of the *B. thailandensis* wild-type E264 strain and Δ*btaR3*, Δ*btaI3*, and Δ*btaI1* Δ*btaI2* Δ*btaI3* mutant strains. Cultures were supplemented with 10 µM 3OHC_8_-HSL. Acetonitrile only was added to the controls. The values represent the means for three replicates. The luminescence is expressed in relative light units per optical density of the culture (RLU/OD_600_). Download FIG S2, PDF file, 0.04 MB. Copyright © 2017 Le Guillouzer et al. 2017 Le Guillouzer et al. This content is distributed under the terms of the Creative Commons Attribution 4.0 International license.

To confirm that the QS-3 system is not exclusively modulated by BtaR3, we investigated the influence of BtaR1 and BtaR2 on 3OHC_8_-HSL biosynthesis and *btaI3* expression. As previously noted for C_8_-HSL and 3OHC_10_-HSL, the levels of 3OHC_8_-HSL were enhanced in the Δ*btaR1* mutant compared to the wild-type strain (Fig. 4A). While 3OHC_10_-HSL overproduction was observed during the different stages of bacterial growth (Fig. 3A), augmentation of 3OHC_8_-HSL concentrations occurred principally in the late exponential phase in the Δ*btaR1* mutant (Fig. 4A). Surprisingly, expression of *btaI3* was lower, suggesting that the negative regulation of 3OHC_8_-HSL biosynthesis by BtaR1 is indirect and does not result from *btaI3* modulation (Fig. 4B). Additionally, we observed an increase in 3OHC_8_-HSL levels in the Δ*btaR*2 mutant from late logarithmic growth (Fig. 4A). Nevertheless, no obvious change in expression of *btaI3* was visible, revealing that BtaR2 might not repress 3OHC_8_-HSL biosynthesis through regulation of *btaI3* transcription as well (Fig. 4B). All in all, these findings demonstrate that the QS-1, QS-2, and QS-3 systems work collectively to regulate production of AHLs.

We also analyzed production of AHLs in the Δ*btaR4* and Δ*btaR5* mutants, and no difference with the wild-type strain production was found, revealing that neither BtaR4 nor BtaR5 was involved in the regulation of the biosynthesis of C_8_-HSL, 3OHC_10_-HSL, and 3OHC_8_-HSL under the conditions of our experiments (data not shown).

### The *btaR1*, *btaR2*, and *btaR3* genes are QS controlled.

**FIG 5:**
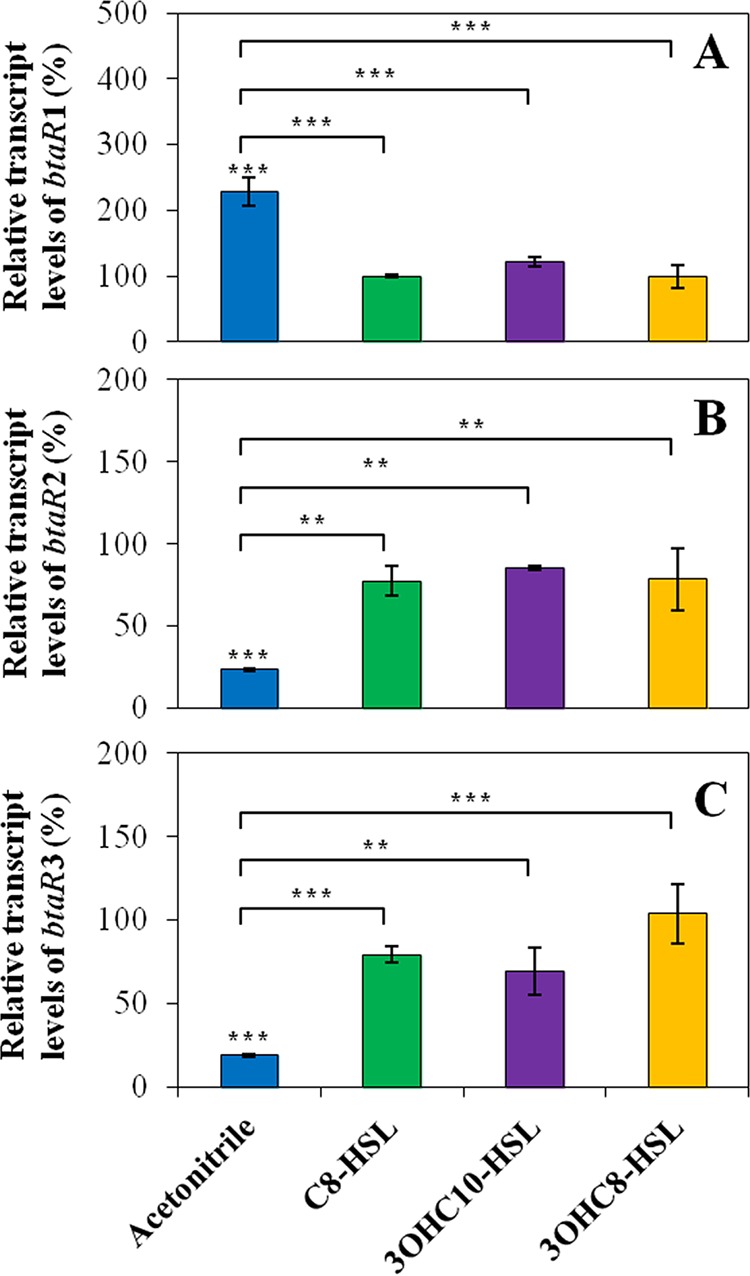
Effects of AHLs on the levels of expression of the *btaR1*, *btaR2*, and *btaR3* genes. The relative transcript levels of (A) *btaR1*, (B) *btaR2*, and (C) *btaR3* from the wild-type *B. thailandensis* E264 strain and its Δ*btaI1* Δ*btaI2* Δ*btaI3* mutant strain were estimated by qRT-PCR. Cultures were supplemented with 10 µM C_8_-HSL, 3OHC_10_-HSL, or 3OHC_8_-HSL. Acetonitrile only was added to the controls. The results are presented as relative quantification of transcription of the gene compared to the wild-type strain, which was set at 100%. The error bars represent the standard deviations of the averages for three replicates. ***, *P* < 0.001; **, *P* < 0.01.

In order to verify whether the QS modulatory cascade also involves cross-regulation between the BtaR transcriptional regulators, the levels of expression of *btaR1*, *btaR2*, and *btaR3* were assessed by quantitative reverse transcription-PCR (qRT-PCR) in the wild-type *B. thailandensis* E264 strain and in the AHL-defective Δ*btaI1* Δ*btaI2* Δ*btaI3* mutant during the exponential phase. Interestingly, the transcription of *btaR1*, *btaR2*, and *btaR3* was significantly affected by the absence of AHLs, indicating that they are controlled by QS (Fig. 5). *btaR1* transcription was increased in the Δ*btaI1* Δ*btaI2* Δ*btaI3* mutant compared to the wild-type strain, revealing that its expression is negatively regulated by AHLs (Fig. 5A). Conversely, *btaR2* and *btaR3* were both downregulated in the absence of AHLs, showing that these genes are activated by QS (Fig. 5B and C). To further investigate the impact of AHLs on the expression of *btaR1*, *btaR2*, and *btaR3*, their transcription was measured in the Δ*btaI1* Δ*btaI2* Δ*btaI3* mutant supplemented with exogenous C_8_-HSL, 3OHC_10_-HSL, or 3OHC_8_-HSL. Interestingly, the levels of expression of *btaR1*, *btaR2*, and *btaR3* were restored to wild-type levels in the presence of AHLs produced by their respective cognate synthase, as well as in the presence of noncognate AHLs, suggesting that their regulation depends on themselves and on other QS systems (Fig. 5). Collectively, our results indicate that the interdependence of the QS-1, QS-2, and QS-3 systems also implicates cross-modulation between BtaR1, BtaR2, and BtaR3.

### The levels of expression of *btaI1*, *btaI2*, and *btaI3* are modulated by cognate and noncognate AHLs.

To further elucidate the regulatory mechanisms directing *btaI1*, *btaI2*, and *btaI3* expression, the activity of the corresponding chromosomal *lux* transcriptional reporters was measured in the AHL-defective Δ*btaI1* Δ*btaI2* Δ*btaI3* mutant supplemented with exogenous AHLs or not supplemented with AHLs. Since we noticed that the QS-1 and QS-2 systems were both activated in the logarithmic growth phase, whereas activation of the QS-3 system started in stationary phase (Fig. 1), experiments with *btaI1*-*lux* and *btaI2*-*lux* were done during the exponential phase, while those with *btaI3*-*lux* were performed during the stationary phase. Additionally, the impact of AHLs on the transcription of *btaI1*, *btaI2*, and *btaI3* was also estimated by monitoring the activity of *btaI1*-*lux*, *btaI2*-*lux*, and *btaI3*-*lux*, respectively, in cultures of the Δ*btaI1*, Δ*btaI2*, and Δ*btaI3* mutants versus the wild-type *B. thailandensis* E264 strain throughout the bacterial growth phases.

**FIG 6:**
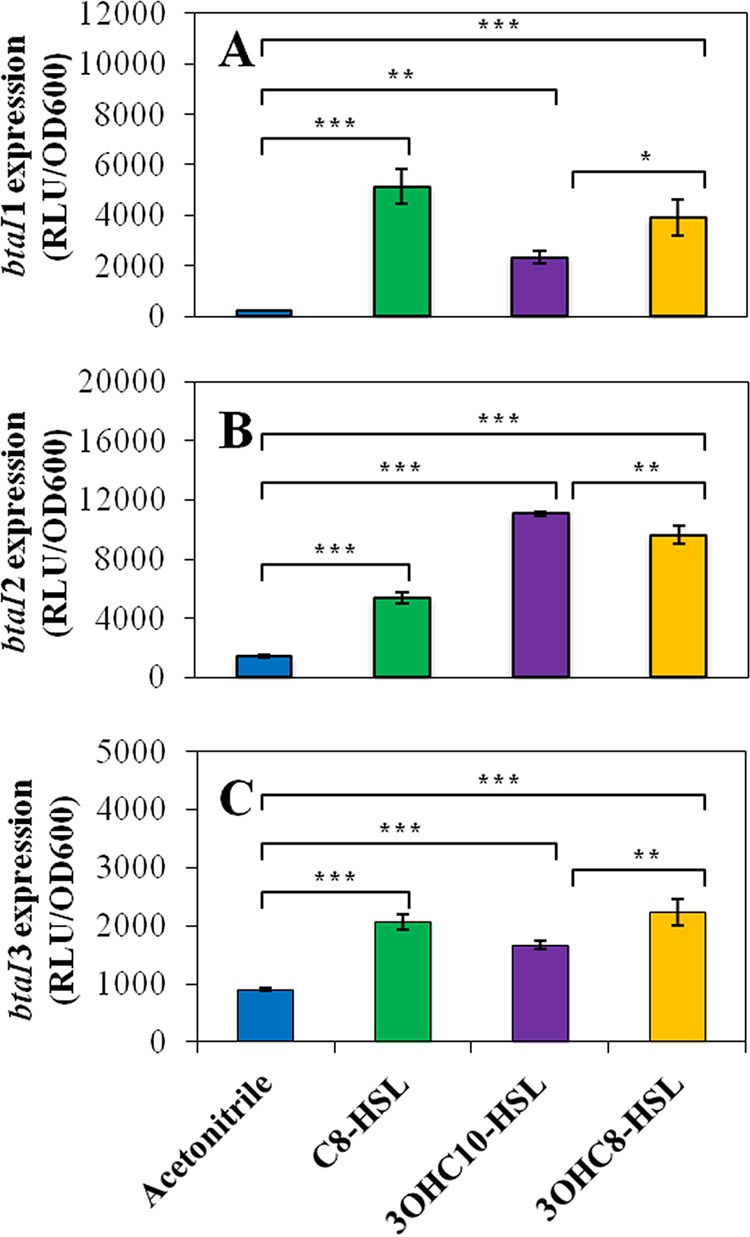
Activation of expression from the *btaI1*, *btaI2*, and *btaI3* promoters by AHLs. The luciferase activity of the chromosomal (A) *btaI1*-*lux*, (B) *btaI2*-*lux*, and (C) *btaI3*-*lux* transcriptional fusions was monitored in cultures of the *B. thailandensis* E264 Δ*btaI1* Δ*btaI2* Δ*btaI3* mutant strain. Cultures were supplemented with 10 µM C_8_-HSL, 3OHC_10_-HSL, or 3OHC_8_-HSL. Acetonitrile only was added to the controls. The error bars represent the standard deviations of the averages for three replicates. The luminescence is expressed in relative light units per optical density of the culture (RLU/OD_600_). ***, *P* < 0.001; **, *P* < 0.01; *, *P* < 0.05.

While we demonstrated that *btaI1* is positively controlled by BtaR1 and activated by BtaI1-produced C_8_-HSL (Fig. S1), expression of *btaI1* was also enhanced in the presence of noncognate AHLs, namely, 3OHC_10_-HSL and 3OHC_8_-HSL (13), in the AHL-negative Δ*btaI1* Δ*btaI2* Δ*btaI3* mutant background (Fig. 6A). Since we found that BtaR3 activates *btaI1* as well (Fig. 2B), we tested the impact of 3OHC_10_-HSL and 3OHC_8_-HSL on *btaI1* transcription in the absence of BtaR3 in order to verify whether activation of *btaI1* by these AHLs could be dependent on BtaR3. No significant effect on *btaI1* transcription was visible in cultures of the Δ*btaR3* mutant supplemented with either 3OHC_10_-HSL or 3OHC_8_-HSL (data not shown). This suggests that BtaR3 is necessary for activation of *btaI1* by these AHLs. Collectively, these observations confirm that *btaI1* is mainly activated by BtaR1/C_8_-HSL and might also be positively regulated by BtaR3 in conjunction with 3OHC_10_-HSL and 3OHC_8_-HSL.

Expression of *btaI2* was more strongly enhanced by 3OHC_10_-HSL (Fig. 6B). We also noticed a significant activation with 3OHC_8_-HSL (Fig. 6B). Surprisingly, activation in the presence of the noncognate C_8_-HSL was observed as well, revealing that expression of *btaI2* is not exclusively under BtaR2 control (Fig. 6B). Additionally, we confirmed that BtaR2 directly modulates *btaI2* transcription in response to 3OHC_10_-HSL and 3OHC_8_-HSL, produced by its cognate synthase BtaI2 (16), but does not function with C_8_-HSL (Fig. S3). Altogether, these data confirm that *btaI2* is positively regulated by BtaR2 in response to both 3OHC_10_-HSL and 3OHC_8_-HSL, whereas activation by C_8_-HSL is independent of BtaR2.

10.1128/mBio.01861-17.3 FIG S3  *btaI2* is directly activated by BtaR2 in response to 3OHC_8_-HSL or 3OHC_10_-HSL. The luciferase activity of the chromosomal *btaI2*-*lux* transcriptional fusion was monitored in the heterologous system, namely, *E. coli* DH5α also containing a BtaR2 expression vector with an arabinose-inducible promoter. Cultures were supplemented with 10 µM C_8_-HSL, 3OHC_8_-HSL, or 3OHC_10_-HSL. Acetonitrile only was added to the controls. The values represent the means for three replicates. The luminescence is expressed in relative light units per optical density of the culture (RLU/OD_600_). Download FIG S3, PDF file, 0.04 MB. Copyright © 2017 Le Guillouzer et al. 2017 Le Guillouzer et al. This content is distributed under the terms of the Creative Commons Attribution 4.0 International license.

**FIG 7:**
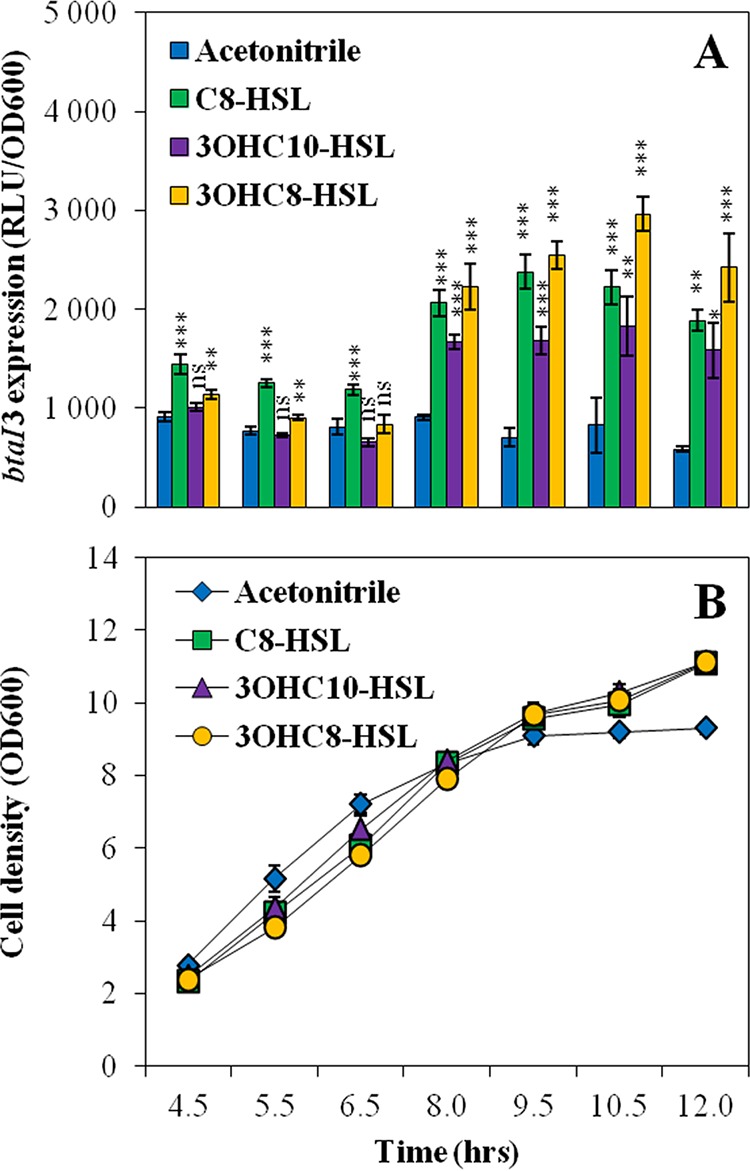
Activation of expression from the *btaI3* promoter by AHLs. (A) The luciferase activity of the chromosomal *btaI3*-*lux* transcriptional fusion was monitored at (B) various times during growth in cultures of the *B. thailandensis* E264 Δ*btaI1* Δ*btaI2* Δ*btaI3* mutant strain. Cultures were supplemented with 10 µM C_8_-HSL, 3OHC_10_-HSL, or 3OHC_8_-HSL. Acetonitrile only was added to the controls. The error bars represent the standard deviations of the averages for three replicates. The luminescence is expressed in relative light units per optical density of the culture (RLU/OD_600_). ***, *P* < 0.001; **, *P* < 0.01; *, *P* < 0.05; ns, nonsignificant.

Expression of *btaI3* was at least doubled in cultures of the Δ*btaI1* Δ*btaI2* Δ*btaI3* mutant when supplemented with any of the three AHLs (Fig. 6C), with 3OHC_8_-HSL being the most efficient AHL. Interestingly, 3OHC_8_-HSL had no impact in the Δ*btaI1* Δ*btaI2* Δ*btaI3* mutant background with coaddition of C_8_-HSL and 3OHC_10_-HSL, suggesting that these AHLs might compete for *btaI3* activation (Fig. S4). Similarly to 3OHC_8_-HSL, the expression of *btaI3* was not enhanced by 3OHC_10_-HSL in the absence of BtaR3 (data not shown), showing that BtaR3 responds to both 3OHC_8_-HSL and 3OHC_10_-HSL to stimulate *btaI3* transcription. Since all three AHLs seem able to activate expression of *btaI3*, we investigated whether their respective influence changes over the various growth phases. Strikingly, *btaI3* was mostly activated by C_8_-HSL during the logarithmic growth phase, whereas activation of *btaI3* by 3OHC_8_-HSL and 3OHC_10_-HSL was more prominent during the stationary phase (Fig. 7). Taken together, these results indicate that *btaI3* is activated by BtaR1/C_8_-HSL in the exponential growth phase and is also positively regulated by BtaR3 in association with 3OHC_8_-HSL and 3OHC_10_-HSL in the stationary phase.

10.1128/mBio.01861-17.4 FIG S4  3OHC_8_-HSL activation of *btaI3* is dependent on C_8_-HSL and 3OHC_10_-HSL. The luciferase activity of the chromosomal *btaI3*-*lux* transcriptional fusion was measured during stationary phase in cultures of the *B. thailandensis* wild-type E264 strain and the Δ*btaI1* Δ*btaI2* Δ*btaI3* mutant strain. Cultures were supplemented with 10 µM C_8_-HSL, 3OHC_8_-HSL, and 3OHC_10_-HSL. Acetonitrile only was added to the controls. The values represent the means for three replicates. The luminescence is expressed in relative light units per optical density of the culture (RLU/OD_600_). Download FIG S4, PDF file, 0.04 MB. Copyright © 2017 Le Guillouzer et al. 2017 Le Guillouzer et al. This content is distributed under the terms of the Creative Commons Attribution 4.0 International license.

## DISCUSSION

Although the QS-1, QS-2, and QS-3 systems of *B. thailandensis* had been previously described (13, 16, 20), a detailed picture of the interactions between the elements composing this complex QS regulatory network was missing. Since the real impact of the BtaR transcriptional regulators on the biosynthesis of their cognate AHLs and expression of adjacent *btaI* genes was assumed in the literature but almost never confirmed experimentally, we investigated production of AHLs in all Δ*btaR* mutants and compared it with measurements of the levels of expression of *btaI* genes.

**FIG 8:**
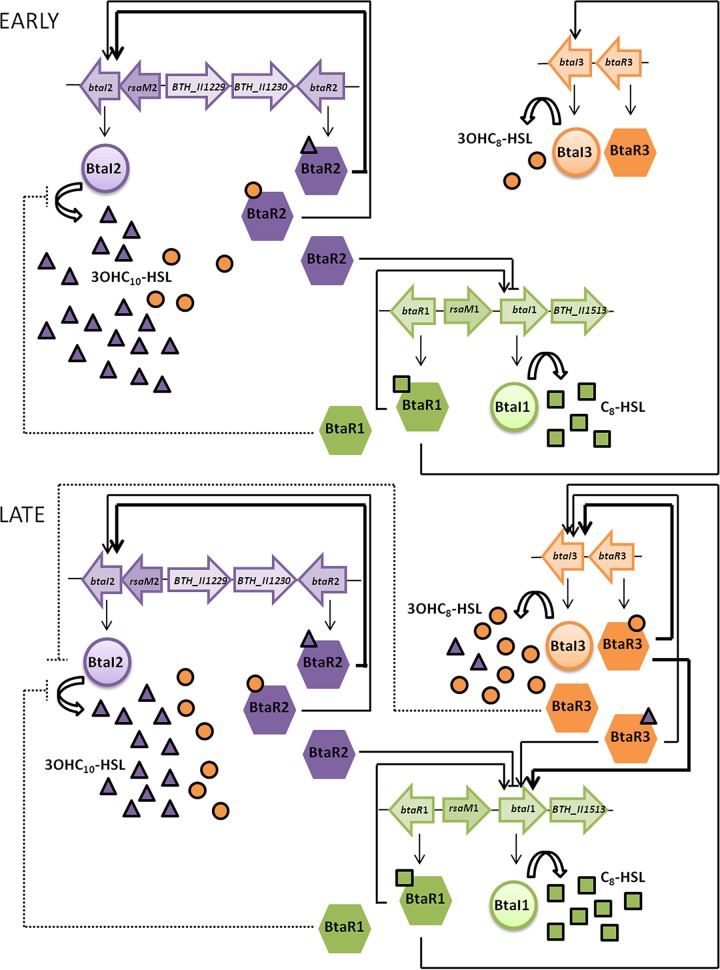
Proposed interactions between the QS-1, QS-2, and QS-3 systems.

As previously described for *B. pseudomallei* KHW (17), we observed variations in the biosynthesis of the main AHLs as well as in the transcription of the AHL synthase-coding genes *btaI1*, *btaI2*, and *btaI3* throughout the growth phases in *B. thailandensis* E264 (Fig. 1). These observations highlighted the timing of expression of the QS-1, QS-2, and QS-3 systems during the different stages of growth and consequently the existence of potential interactions between these QS circuits. While C_8_-HSL is generally considered the primary AHL produced by *Burkholderia* spp. (4) and is indeed predominately detected in stationary-phase cultures of *B. pseudomallei* K96243 and *B. mallei* ATCC 23344 (15, 17), we confirmed that 3OHC_10_-HSL is actually the most abundant AHL found in *B. thailandensis* E264 cultures during the different stages of growth, revealing the importance of the QS-2 system in the QS circuitry of *B. thailandensis* E264 (Fig. 8).

While we confirmed that transcription of *btaI2* and biosynthesis of 3OHC_10_-HSL are activated by BtaR2, a stronger activation by 3OHC_10_-HSL indicates that BtaR2 exhibits higher affinity for this AHL than for 3OHC_8_-HSL (Fig. 6B), which is also produced by the same synthase (16). Similarly, the *bpsI2* gene that codes for the BpsI2 synthase was also shown to be substantially enhanced by 3OHC_10_-HSL in *B. pseudomallei* KHW (17). The fact remains that the levels of expression of *btaI2* were similar in the wild-type E264 strain of *B. thailandensis* and in the non-3OHC_10_-HSL-producing Δ*btaI2* mutant (Fig. 3B). Considering that 3OHC_8_-HSL is still produced in the absence of BtaI2 (16), we must conclude that both 3OHC_10_-HSL and 3OHC_8_-HSL can induce the transcription of *btaI2* (Fig. 8). Because we confirmed that BtaR2 does not function with C_8_-HSL (Fig. S3), an alternative LuxR-type transcriptional regulator is likely involved in its effect on *btaI2* expression, highlighting an interaction between the QS-1 and QS-2 systems.

Although both BtaR1 and BtaR3 affect 3OHC_10_-HSL production (Fig. 3A), indicating that regulation of the biosynthesis of this AHL implies dynamic coordination between the *B. thailandensis* E264 QS-1, QS-2, and QS-3 circuits (Fig. 8), neither one has an effect on *btaI2* expression (Fig. 3B). Nevertheless, Majerczyk et al. (20) demonstrated that *btaR2* expression is stimulated by 3OHC_8_-HSL, and we determined that the transcription of this gene is in fact affected by the absence of all AHLs found in *B. thailandensis* E264 (Fig. 5B). Thus, we hypothesize that BtaR1 and BtaR3 act indirectly through *btaR2* control. We also do not exclude the possibility that additional transcriptional and/or posttranscriptional regulators are involved in the modulation of the QS-2 system. Interestingly, this system contains an additional gene between *btaI2* and *btaR2* that is conserved in the *Burkholderia* genus (21). It encodes a hypothetical protein that is 37% identical to the *B. cenocepacia* J2315 *Bc*RsaM (22), a homologue of the QS repressor RsaM originally identified in the plant pathogen *Pseudomonas fuscovaginae* (23), which we consequently renamed RsaM2 (Fig. S5). Accordingly, we observed that C_8_-HSL, 3OHC_10_-HSL, and 3OHC_8_-HSL concentrations were all increased in an *rsaM2* mutant compared to the wild-type strain (24), indicating that RsaM2 likely intervenes in the regulation of all QS systems of *B. thailandensis* E264.

10.1128/mBio.01861-17.5 FIG S5  Genetic organization of the QS regulatory genes in *B. thailandensis* E264. *btaI1* and *btaR1* are not located next to each other and are divergently transcribed in *B. thailandensis* E264. The promoter region of *btaI1* contains a putative *lux* box sequence centered 73.5 bp upstream of the *btaI1* translation start site (CCCTGTAAGGGTTAACAGTT). *btaI2* and *btaR*2 are also not located next to each other and are transcribed in the same direction on the genome of *B. thailandensis* E264. The promoter region of *btaI2* contains a putative *lux* box sequence centered 65.0 bp upstream of the *btaI2* translation start site (ACCTGTAGAAATCGTAGT). *btaI3* and *btaR3* are also transcribed in the same direction and are located next to each other in *B. thailandensis* E264. Download FIG S5, PDF file, 0.1 MB. Copyright © 2017 Le Guillouzer et al. 2017 Le Guillouzer et al. This content is distributed under the terms of the Creative Commons Attribution 4.0 International license.

As described previously for the *B. pseudomallei* KHW BpsI and *B. mallei* ATCC 23344 BmaI1 synthases (11, 15), Chandler et al. (13) demonstrated that BtaI1 is responsible for C_8_-HSL production. In agreement with the finding that the *B. pseudomallei* K96243 BpsR and *B. mallei* ATCC 23344 BmaR1 transcriptional regulators directly activate the BpsI- and BmaI1-encoding genes in response to C_8_-HSL, respectively (15, 25), Majerczyk et al. (20) reported that *btaI1* transcription is positively modulated by BtaR1. We observed a strong BtaR1-dependent induction of *btaI1* through C_8_-HSL (Fig. S1) and confirmed that the QS-1 system responds best toward its cognate AHL (Fig. 6A). While we demonstrated that BtaR1 constitutes the main regulator of *btaI1* expression, we assume that BtaR1 represents the main regulator of C_8_-HSL biosynthesis as well. An uncoupling of AHL production and expression of the corresponding synthase was also reported in a *Burkholderia* RsaM-deficient strain (22, 26). *Bc*RsaM from *B. cenocepacia* H111 was indeed described as an important repressor of C_8_-HSL biosynthesis and shown to activate the transcription of *cepI* and *cepR* encoding the LuxI-type synthase CepI and the LuxR-type transcriptional regulator CepR, respectively (22, 26). Interestingly, a gene encoding a hypothetical protein sharing 63% identity with the *B. cenocepacia* J2315 *Bc*RsaM, hence called RsaM1, was also found between *btaI1* and *btaR1* (Fig. S5). Investigating the effect of RsaM1 on the biosynthesis of AHLs in *B. thailandensis* E264 showed that C_8_-HSL is overproduced in an *rsaM1* mutant compared to the wild-type strain (24), revealing a possible link between the QS-1 system and RsaM1. Additional experiments will be necessary to fully understand the mechanisms involved in the regulation of the QS-1 system as well as the implications of the RsaM-like proteins in *B. thailandensis* E264.

We demonstrated that the biosynthesis of C_8_-HSL and transcription of *btaI1* are both negatively controlled by BtaR2 (Fig. 2). Because no overexpression of the *btaI1* gene was observed in the Δ*btaI2* mutant background, we assume that BtaR2 represses the QS-1 system in the absence of its ligands. This contrasts with the BtaR3-dependent regulation of *btaI1* transcription in conjunction with 3OHC_8_-HSL, as well as with 3OHC_10_-HSL, albeit to a lesser extent (Fig. 8). This is also further supported by the fact that BpsR3 was reported to directly activate *bpsI* in response to both 3OHC_8_-HSL and 3OHC_10_-HSL, with 3OHC_8_-HSL eliciting the strongest response from BpsR3 (17). Considering that *bmaI1* was also shown to be directly controlled by BmaR3/3OHC_8_-HSL (14), we suppose that BtaR3 directly activates expression of the *btaI1* gene as well. However, we believe the effect of BtaR3 on the QS-1 system is more complex. While the *bpsR* gene encoding BpsR was reported to be positively autoregulated (11), we determined that *btaR1* expression is repressed by QS (Fig. 5A). Thus, negative regulation of C_8_-HSL biosynthesis by BtaR3 could be linked to *btaR1* modulation. Altogether, these observations further highlight the existence of interactions between the QS-1, QS-2, and QS-3 circuits and reveal that the timing of expression of the QS-1 system is dependent on both the QS-2 and QS-3 systems (Fig. 8). This might contribute to the successive activation of the *B. thailandensis* E264 QS circuits observed throughout bacterial growth.

Similarly to the *B. pseudomallei* KWH BpsI3 and *B. mallei* ATCC 23344 BmaI3 synthases, BtaI3 was shown to produce 3OHC_8_-HSL (13, 14, 17). While the *B. pseudomallei* KHW BpsR3 and *B. mallei* ATCC 23344 BmaR3 transcriptional regulators specifically respond to 3OHC_8_-HSL, the *bpsI3* and *bmaI3* genes were not reported to be activated by BpsR3 and BmaR3, respectively, in conjunction with 3OHC_8_-HSL (14, 17). Here, in *B. thailandensis* E264, we demonstrated that the transcription of *btaI3* is positively controlled by BtaR3 and activated by 3OHC_8_-HSL (Fig. S2). However, 3OHC_8_-HSL-dependent activation of *btaI3* seems to be conditioned by the presence of other AHLs (Fig. S4). The interaction between BtaR3 and 3OHC_8_-HSL, necessary to activate *btaI3* expression, could be impeded by a competitive inhibition exerted by another AHL, as already proposed for *B. pseudomallei* KHW (17). In addition, we observed that *btaI3* expression is activated by 3OHC_10_-HSL, albeit to a lesser extent (Fig. 6C). Indeed, the BtaR3-controlled genes identified in transcriptomic analyses were also generally affected by both 3OHC_8_-HSL and 3OHC_10_-HSL (20). This further supports the idea that BtaR3 functions with these two AHLs (Fig. 8). Considering that BpsI3 and BmaI3 were both shown to produce 3OHC_10_-HSL in addition to 3OHC_8_-HSL (14, 17), it is possible that BtaI3 intervenes in the biosynthesis of 3OHC_10_-HSL in *B. thailandensis* E264 as well.

Remarkably, positive 3OHC_8_-HSL- and 3OHC_10_-HSL-dependent regulation of *btaI3* occurred in the stationary growth phase (Fig. 7), in agreement with the expression profile of this gene. Conversely, activation of *btaI2* transcription by these AHLs was mainly observed during logarithmic growth. We thus hypothesize that the QS-3 system regulates the QS-2 system targets by producing 3OHC_8_-HSL in stationary phase, whereas production of this AHL by the QS-2 system occurs essentially during the exponential phase, implying a coordination between the QS-2 and QS-3 systems (Fig. 8). Additionally, it seems that 3OHC_8_-HSL is produced by BtaI2 at the expense of 3OHC_10_-HSL. This would explain why there is an overlap between these QS circuits when it comes to genes modulated by 3OHC_8_-HSL and 3OHC_10_-HSL (20). Importantly, while sharing common AHLs, the QS-2 and QS-3 systems are apparently not transcriptionally linked.

The BtaR1/C_8_-HSL-dependent control of *btaI3* transcription, which starts in the exponential growth phase, is consistent with the idea that the QS-1 system is required for the expression of *btaI3* (20), and might also account for the belated activation of the QS-3 circuit in comparison with the QS-1 and QS-2 systems. This again illustrates the successive expression of these QS circuits and points toward an interdependence between the QS-1 and QS-3 systems (Fig. 8). Such an interconnection has already been observed among the members of the *Bptm* group, as *bpsI3* transcription was reported to be stimulated by the BpsI/BpsR QS system (17). Nevertheless, the precise regulatory mechanism directing the QS-3 system through BtaR1 is currently unknown. While BtaR1 seems to act by activating *btaI3* transcription, we propose that the negative impact of BtaR1 on 3OHC_8_-HSL biosynthesis does not result from a direct interaction with the *btaI3* promoter but rather could imply the effect of BtaR1 on the level of *btaR3* as previously suggested (20). Additional transcriptional and/or posttranscriptional regulators might also be involved in the BtaR1-dependent modulation of the QS-3 system.

### Conclusion.

The study described here provides for the first time an exhaustive portrait of the interplay between the QS-1, QS-2, and QS-3 systems in *B. thailandensis* E264 (Fig. 8). We observed an interdependence between the QS-1 and QS-2 systems. While we confirmed that the QS-3 system is controlled by BtaR1, we also found that BtaR3 modulates the QS-1 system, which indicates that those two systems are linked. Interestingly, such an interaction between the QS-1 and QS-3 systems seems to be conserved in the closely related species of the *Bptm* group (14, 17, 20). Interestingly, the QS-2 and QS-3 systems that share common AHLs seem not to be transcriptionally linked, but instead they are temporally connected by their common AHLs. We also highlighted a surprising uncoupling of AHL production and expression of the corresponding synthase in the QS-1 system, which hints that QS regulation does not always follow a classic pattern. Collectively, the results of our study suggest that there are homeostatic regulatory loops provided by the various QS systems in *B. thailandensis* resulting from transcriptional and posttranscriptional interactions, allowing tightly controlled coordination of the expression of genes.

Although we have found new connections and insights on the QS cascade, there are still many questions to be answered. Indeed, further work is needed to comprehend more about the mechanisms behind those links and regulation as well as the implications of recently characterized RsaM-like proteins. The temporal pattern of QS-controlled genes clearly shows that additional factors are involved (17, 20, 27).

## MATERIALS AND METHODS

### Bacterial strains and culture conditions.

The bacterial strains used in this study are listed in Table S1 in the supplemental material. Unless stated otherwise, all bacteria were cultured at 37°C in tryptic soy broth (TSB) (BD Difco, Mississauga, Ontario, Canada), with shaking (240 rpm) in a TC-7 roller drum (New Brunswick, Canada), or on petri dishes containing TSB solidified with 1.5% agar. When required, antibiotics were used at the following concentrations: 15 µg/ml tetracycline (Tc) and 25 µg/ml gentamicin (Gm) for *Escherichia coli* DH5α, while Tc was used at 200 µg/ml for *Burkholderia  thailandensis* E264. All measurements of optical density (optical density at 600 nm [OD_600_]) were acquired with a Thermo Fisher Scientific NanoDrop ND-1000 spectrophotometer.

10.1128/mBio.01861-17.6 TABLE S1  Bacterial strains used in this study. Download TABLE S1, DOCX file, 0.02 MB. Copyright © 2017 Le Guillouzer et al. 2017 Le Guillouzer et al. This content is distributed under the terms of the Creative Commons Attribution 4.0 International license.

### Construction of plasmids.

All plasmids used in this study are described in Table S2. Amplification of the promoter regions of *btaI1*, *btaI2*, and *btaI3* was performed from genomic DNA from *B. thailandensis* E264 using the appropriate primers (Table S3). The amplified products were digested with the FastDigest restriction enzymes XhoI and BamHI (Thermo Fisher Scientific) and inserted by T4 DNA ligase (Bio Basic, Inc., Markham, ON, Canada) within the corresponding restriction sites in the mini-CTX-*lux* plasmid (28), generating the transcriptional reporters pSLG02, pSLG03, and pSLG04, respectively. All primers were from Alpha DNA (Montreal, Quebec, Canada).

10.1128/mBio.01861-17.7 TABLE S2  Plasmids used in this study. Download TABLE S2, DOCX file, 0.02 MB. Copyright © 2017 Le Guillouzer et al. 2017 Le Guillouzer et al. This content is distributed under the terms of the Creative Commons Attribution 4.0 International license.

10.1128/mBio.01861-17.8 TABLE S3  Primers used for PCR. Download TABLE S3, DOCX file, 0.01 MB. Copyright © 2017 Le Guillouzer et al. 2017 Le Guillouzer et al. This content is distributed under the terms of the Creative Commons Attribution 4.0 International license.

### Construction of reporter strains.

The mini-CTX-*btaI1*-*lux*, mini-CTX-*btaI2*-*lux*, and mini-CTX-*btaI3*-*lux* transcriptional reporters were integrated into the chromosomes of *B. thailandensis* E264 strains through conjugation with *E. coli* χ7213 followed by selection with Tc. Successful chromosomal insertion of the *btaI1*-*lux*, *btaI2*-*lux*, and *btaI3*-*lux* plasmids was confirmed by PCR using the appropriate primers.

### LC-MS/MS quantification of AHLs.

The concentrations of AHLs were determined from samples of *B. thailandensis* E264 cultures obtained at different time points during bacterial growth, by liquid chromatography coupled to tandem mass spectrometry (LC-MS/MS). The samples were prepared and analyzed as described previously (29). 5,6,7,8-Tetradeutero-4-hydroxy-2-heptylquinoline (HHQ-d4) was used as an internal standard. All experiments were performed in triplicate and conducted at least twice independently.

### Measurement of the activity of *btaI1*-*lux*, *btaI2*-*lux*, and *btaI3*-*lux* reporters.

The levels of expression from the promoter regions of *btaI1*, *btaI2*, or *btaI3* were quantified by measuring the luminescence of *B. thailandensis* E264 cultures carrying the corresponding chromosomal reporters. Overnight bacterial cultures were diluted in TSB to an initial OD_600_ of 0.1 and incubated as described above. The luminescence was regularly determined from culture samples using a multimode microplate reader (Cytation 3; Bio-Tek Instruments, Inc., Winooski, VT, USA) and expressed in relative light units per optical density of the culture (RLU/OD_600_). For experiments with AHL additions, cultures were supplemented with 10 µM C_8_-HSL, 3OHC_8_-HSL, and 3OHC_10_-HSL (Sigma-Aldrich Co., Oakville, ON, Canada) or not supplemented with AHLs from stocks prepared in HPLC-grade acetonitrile. Acetonitrile only was added to the controls. All experiments were performed with three biological replicates and repeated at least twice.

### Heterologous *E. coli* expression system for BtaR2 regulation of *btaI2* expression.

The response of the *btaI2* promoter to the BtaR2 transcriptional regulator was determined using a recombinant *E. coli* DH5α strain containing both the chromosomal *btaI2*-*lux* transcriptional fusion and the arabinose-inducible expression vector pJN105-*btaR*2. Overnight bacterial cultures of *E. coli* DH5α were diluted in lysogeny broth (LB) (Alpha Biosciences, Inc., Baltimore, MD) with the appropriate antibiotics and grown in triplicate at 37°C, with shaking in a TC-7 roller drum. When the cultures reached an OD_600_ of 0.5, they were supplemented with 10 μM C_8_-HSL, 3OHC_8_-HSL, or 3OHC_10_-HSL. Acetonitrile only was added to the controls. The BtaR2 expression vector was induced with 0.2% l-arabinose (wt/vol). The *btaI2*-*lux* luciferase activity was measured every 30 min during 10 h as described above. All experiments were repeated at least three times.

### Quantitative reverse transcription-PCR experiments.

Total RNA from *B. thailandensis* E264 cultures at an OD_600_ of 4.0 was extracted with the PureZOL RNA isolation reagent (Bio-Rad Laboratories, Mississauga, ON, Canada) and treated twice with the TURBO DNA-Free kit (Ambion Life Technologies, Inc., Burlington, ON, Canada) according to the manufacturer’s instructions. Extractions were done on three different bacterial cultures. Quality and purity controls were confirmed by agarose gel electrophoresis and UV spectrophotometric analysis, respectively. cDNA synthesis was performed using the iScript reverse transcription supermix (Bio-Rad Laboratories), and amplification was accomplished on a Corbett Life Science Rotor-Gene 6000 thermal cycler using the SsoAdvanced universal SYBR green supermix (Bio-Rad Laboratories), according to the manufacturer’s protocol. The reference gene was *ndh* (30). The *ndh* gene displayed stable expression under the different genetic contexts tested. All primers used for cDNA amplification are presented in Table S4. Differences in gene expression between *Burkholderia thailandensis* E264 strains were calculated using the 2^−ΔΔCT^ formula (31). A threshold of 0.5 was chosen as significant. All experiments were performed in triplicate and conducted at least twice independently.

10.1128/mBio.01861-17.9 TABLE S4  Primers used for qRT-PCR. Download TABLE S4, DOCX file, 0.01 MB. Copyright © 2017 Le Guillouzer et al. 2017 Le Guillouzer et al. This content is distributed under the terms of the Creative Commons Attribution 4.0 International license.

### Data analysis.

Unless stated otherwise, data are reported as means ± standard deviations (SD). Statistical analyses were performed with the R software version 3.3.3 (http://www.R-project.org.) using one-way analysis of variance (ANOVA). Probability values of less than 0.05 were considered significant.
