# Supplementary material for: The Complex Quorum Sensing Circuitry of Burkholderia thailandensis Is Both Hierarchically and Homeostatically Organized
Source: mBio. 2017 Dec 5;8(6):e01861-17. doi: 10.1128/mBio.01861-17 (PMC5717390; doi:10.1128/mBio.01861-17)
Supplement: FIG S1 [file mbo006173620sf1.pdf]

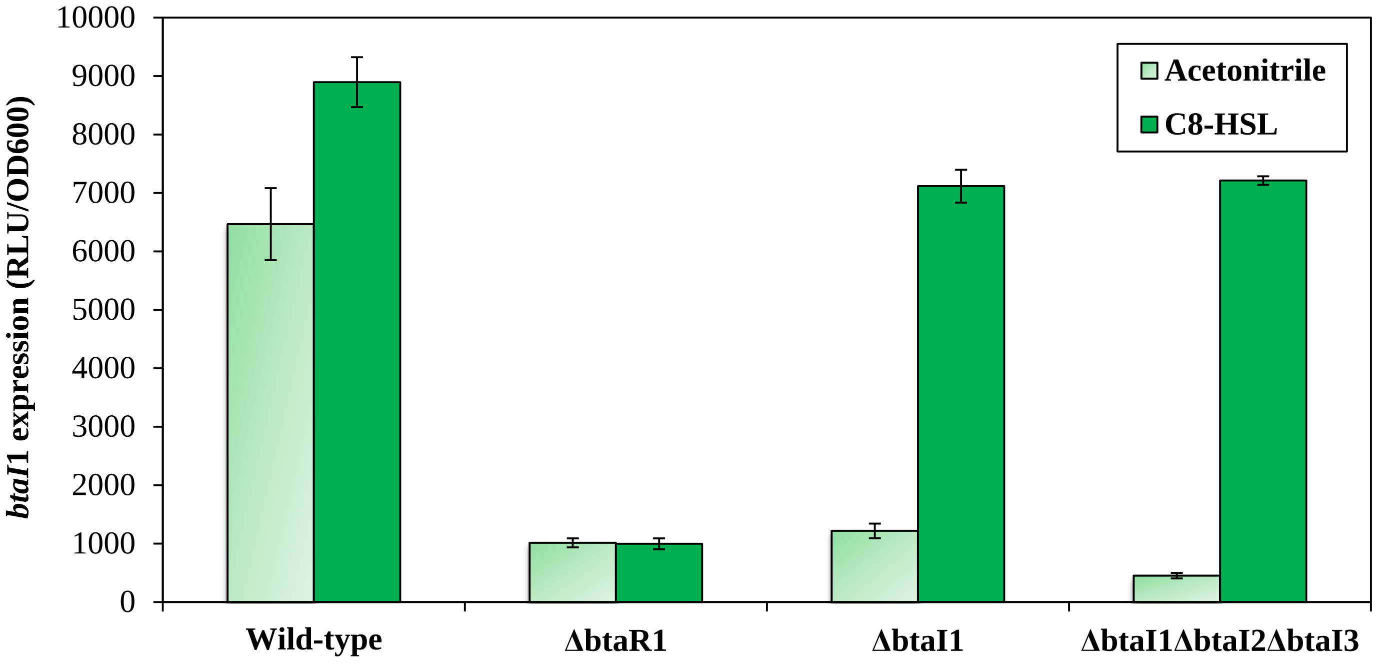

Fig. S1. *btaI*1 activation requires BtaR1 and C_8-HSL. The luciferase activity of the chromosomal *btaI*1-*lux* transcriptional fusion was monitored during the exponential phase in cultures of the *B. thailandensis* E264 wild-type and the Δ*btaR*1, Δ*btaI*1, and Δ*btaI*1Δ*btaI*2Δ*btaI*3 mutant strains. Cultures were supplemented with 10 µM C_8-HSL. Acetonitrile only was added in controls. The values represent the mean of three replicates. The luminescence is expressed in relative light units per culture optical density (RLU/OD_{600}).
