# Supplementary material for: The Complex Quorum Sensing Circuitry of Burkholderia thailandensis Is Both Hierarchically and Homeostatically Organized
Source: mBio. 2017 Dec 5;8(6):e01861-17. doi: 10.1128/mBio.01861-17 (PMC5717390; doi:10.1128/mBio.01861-17)
Supplement: FIG S2 [file mbo006173620sf2.pdf]

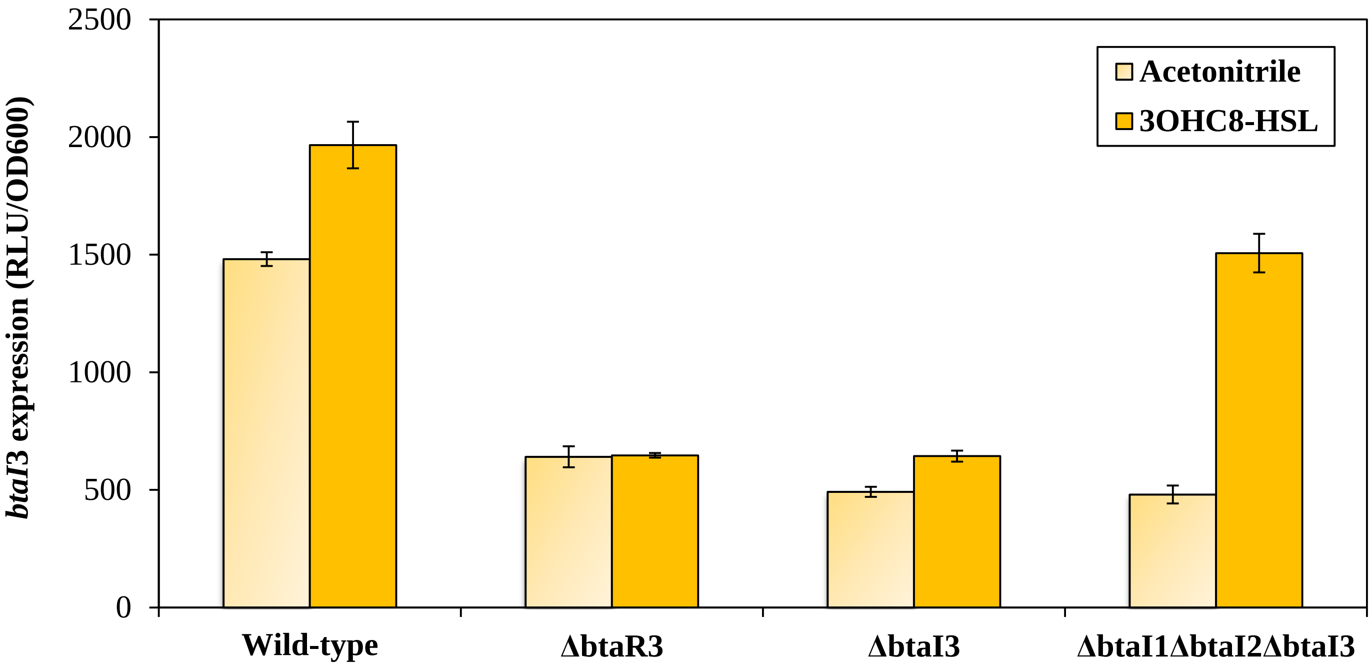

Fig. S2. *btaI*3 is activated by BtaR3 and 3OHC$_8$-HSL. The luciferase activity of the chromosomal *btaI*3-*lux* transcriptional fusion was measured during the stationary phase in cultures of the *B. thailandensis* E264 wild-type and the Δ*btaR*3, Δ*btaI*3, and Δ*btaI*1Δ*btaI*2Δ*btaI*3 mutant strains. Cultures were supplemented with 10 µM 3OHC$_8$-HSL. Acetonitrile only was added in controls. The values represent the mean of three replicates. The luminescence is expressed in relative light units per culture optical density (RLU/OD$_{600}$).
