# Supplementary material for: The Complex Quorum Sensing Circuitry of Burkholderia thailandensis Is Both Hierarchically and Homeostatically Organized
Source: mBio. 2017 Dec 5;8(6):e01861-17. doi: 10.1128/mBio.01861-17 (PMC5717390; doi:10.1128/mBio.01861-17)
Supplement: FIG S3 [file mbo006173620sf3.pdf]

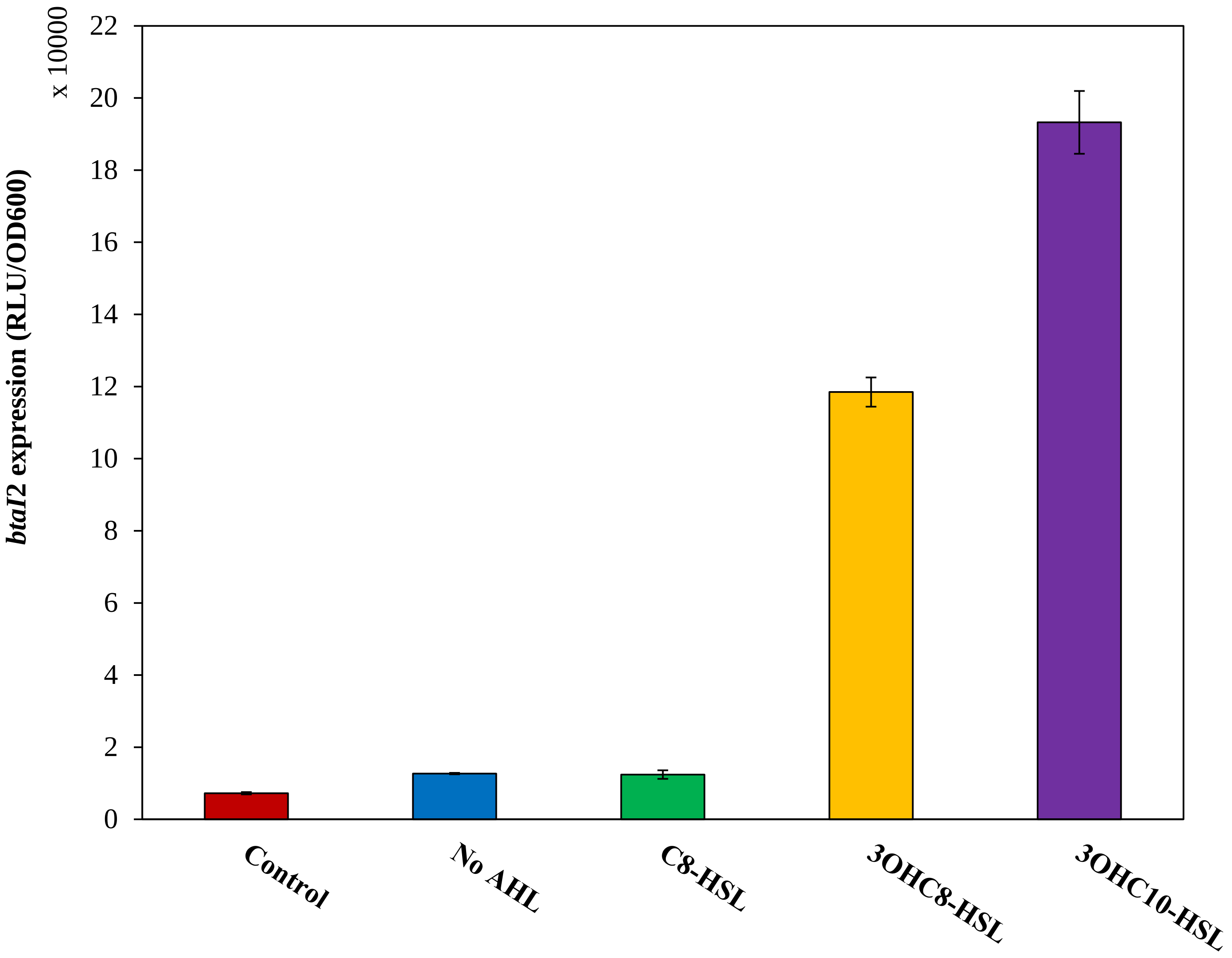

Fig. S3. *btaI*2 is directly activated by BtaR2 in response to 3OHC$_8$-HSL or 3OHC$_{10}$-HSL. The luciferase activity of the chromosomal *btaI*2-*lux* transcriptional fusion was monitored in the heterologous system *E. coli* DH5α also containing a BtaR2 expression vector with an arabinose-inducible promoter. Cultures were supplemented with 10 µM C$_8$-HSL, 3OHC$_8$-HSL, or 3OHC$_{10}$-HSL. Acetonitrile only was added in controls. The values represent the mean of three replicates. The luminescence is expressed in relative light units per culture optical density (RLU/OD$_{600}$).
