# Supplementary material for: The Complex Quorum Sensing Circuitry of Burkholderia thailandensis Is Both Hierarchically and Homeostatically Organized
Source: mBio. 2017 Dec 5;8(6):e01861-17. doi: 10.1128/mBio.01861-17 (PMC5717390; doi:10.1128/mBio.01861-17)
Supplement: FIG S5 [file mbo006173620sf5.pdf]

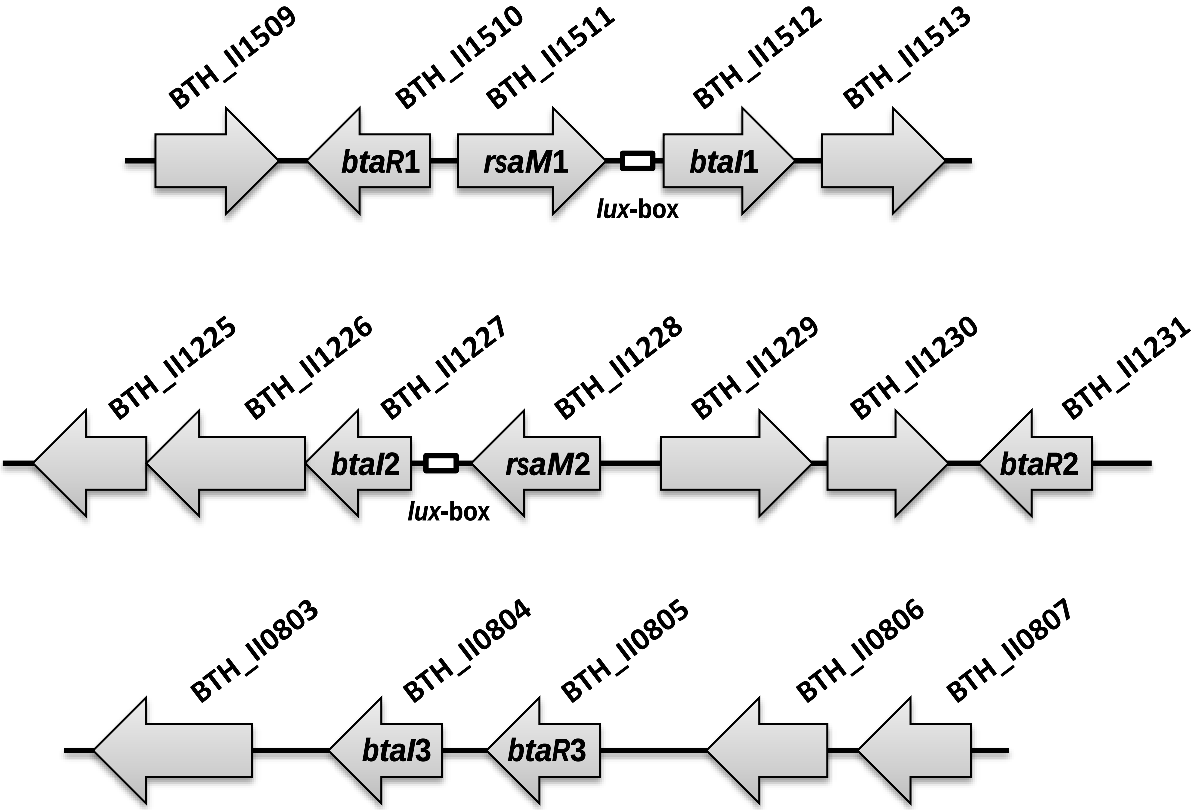

Fig. S5. Genetic organization of the QS regulatory genes in *B. thailandensis* E264. *btaI*1 and *btaR*1 are not located next to each other and are divergently transcribed in *B. thailandensis* E264. The promoter region of *btaI*1 contains a putative *lux*-box sequence centered 73.5 bp usptream of the *btaI*1 translation start site (CCCTGTAAGGGTTAACAGTT). *btaI*2 and *btaR*2 are also not located next to each other and are transcribed in the same direction on the genome of *B. thailandensis* E264. The promoter region of *btaI*2 contains a putative *lux*-box sequence centered 65.0 bp usptream of the *btaI*2 translation start site (ACCTGTAGAAATCGTAGT). *btaI*3 and *btaR*3 are also transcribed in the same direction and are located next to each other in *B. thailandensis* E264.
