# Supplementary material for: The Complex Quorum Sensing Circuitry of Burkholderia thailandensis Is Both Hierarchically and Homeostatically Organized
Source: mBio. 2017 Dec 5;8(6):e01861-17. doi: 10.1128/mBio.01861-17 (PMC5717390; doi:10.1128/mBio.01861-17)
Supplement: TABLE S1 [file mbo006173620st1.docx]

**Table S1. Bacterial strains used in this study.**

| **Strains** | **Description** | **Reference** |
| --- | --- | --- |
| ***E. coli*** |  |  |
| **χ7213** | *thr-*1, *leuB*6, *fhuA*21, *lacY*1, *glnV*44, *recA*1, Δ*asdA*4, Δ(*zhf-*2::Tn*10*)*,* *thi-*1, RP4-2-Tc :: Mu [λ *pir*] | Lab collection |
| **DH5α** | F-, ϕ80d*lacZ*ΔM15, Δ(*lacZYA-argF*)U169, *deoR*, *recA*1, *endA*1, *hsdR*17(rk-, mk+), *phoA*, *supE*44, λ-, *thi-*1, *gyrA*96, *relA*1 | Lab collection |
| **ED3364** | DH5α (pSLG03, pJNR2); Tc^R^, Gm^R^ | This study |
| ***B. thailandensis*** |  |  |
| **E264** | Wild-type | (12) |
| **JBT107** | E264 Δ*btaR*1 | (13) |
| **JBT108** | E264 Δ*btaR*2 | (13) |
| **JBT109** | E264 Δ*btaR*3 | (13) |
| **JBT101** | E264 Δ*btaI*1 | (13) |
| **JBT102** | E264 Δ*btaI*2 | (13) |
| **JBT103** | E264 Δ*btaI*3 | (13) |
| **JBT112** | E264 Δ*btaI*1 Δ*btaI*2 Δ*btaI*3 | (13) |
| **ED3345** | E264 Δ*btaR*1::*btaI*1-*lux* | This study |
| **ED3346** | E264 Δ*btaR*1::*btaI*2-*lux* | This study |
| **ED3347** | E264 Δ*btaR*1::*btaI*3-*lux* | This study |
| **ED3348** | E264 Δ*btaR*2::*btaI*1-*lux* | This study |
| **ED3349** | E264 Δ*btaR*2::*btaI*2-*lux* | This study |
| **ED3350** | E264 Δ*btaR*2::*btaI*3-*lux* | This study |
| **ED3351** | E264 Δ*btaR*3::*btaI*1-*lux* | This study |
| **ED3352** | E264 Δ*btaR*3::*btaI*2-*lux* | This study |
| **ED3353** | E264 Δ*btaR*3::*btaI*3-*lux* | This study |
| **ED3336** | E264 Δ*btaI*1::*btaI*1-*lux* | This study |
| **ED3337** | E264 Δ*btaI*1::*btaI*2-*lux* | This study |
| **ED3338** | E264 Δ*btaI*1::*btaI*3-*lux* | This study |
| **ED3339** | E264 Δ*btaI*2::*btaI*1-*lux* | This study |
| **ED3340** | E264 Δ*btaI*2::*btaI*2-*lux* | This study |
| **ED3341** | E264 Δ*btaI*2::*btaI*3-*lux* | This study |
| **ED3342** | E264 Δ*btaI*3::*btaI*1-*lux* | This study |
| **ED3343** | E264 Δ*btaI*3::*btaI*2-*lux* | This study |
| **ED3344** | E264 Δ*btaI*3::*btaI*3-*lux* | This study |
| **ED3330** | E264::*btaI*1-*lux* | This study |
| **ED3331** | E264::*btaI*2-*lux* | This study |
| **ED3332** | E264::*btaI*3-*lux* | This study |
| **ED3333** | E264 Δ*btaI*1 Δ*btaI*2 Δ*btaI*3::*btaI*1-*lux* | This study |
| **ED3334** | E264 Δ*btaI*1 Δ*btaI*2 Δ*btaI*3::*btaI*2-*lux* | This study |
| **ED3335** | E264 Δ*btaI*1 Δ*btaI*2 Δ*btaI*3::*btaI*3-*lux* | This study |
