# Supplementary material for: The Complex Quorum Sensing Circuitry of Burkholderia thailandensis Is Both Hierarchically and Homeostatically Organized
Source: mBio. 2017 Dec 5;8(6):e01861-17. doi: 10.1128/mBio.01861-17 (PMC5717390; doi:10.1128/mBio.01861-17)
Supplement: TABLE S2 [file mbo006173620st2.docx]

**Table S2. Plasmids used in this study.**

| **Plasmids** | **Description** | **Source** |
| --- | --- | --- |
| **mini-CTX-*lux*** | Integration vector with promoterless *luxCDABE*; Tc^R^ | (28) |
| **pSLG02** | *btaI*1 promoter inserted in *Xho*I-*Bam*HI restriction sites in mini-CTX-*lux*; Tc^R^ | This study |
| **pSLG03** | *btaI*2 promoter inserted in *Xho*I-*Bam*HI restriction sites in mini-CTX-*lux*; Tc^R^ | This study |
| **pSLG04** | *btaI*3 promoter inserted in *Xho*I-*Bam*HI restriction sites in mini-CTX-*lux*; Tc^R^ | This study |
| **pJNR2** | *btaR*2 inserted in *Pst*I-*Sac*I restriction sites in pJN105; Gm^R^ | (16) |
