# Supplementary material for: The Complex Quorum Sensing Circuitry of Burkholderia thailandensis Is Both Hierarchically and Homeostatically Organized
Source: mBio. 2017 Dec 5;8(6):e01861-17. doi: 10.1128/mBio.01861-17 (PMC5717390; doi:10.1128/mBio.01861-17)
Supplement: TABLE S3 [file mbo006173620st3.docx]

**Table S3. Primers used for PCR.**

| **Genes** | **Oligonucleotides** | **Sequences (5’ to 3’)** |
| --- | --- | --- |
| ***btaI*1** | PBtaI1F | CGC**CTCGAG**AACCTGATGGGCATCGAC |
|  | PBtaI1R | CGC**GGATCC**GTCGCCATGAACGAAAGTT |
| ***btaI*2** | PBtaI2F | CGC**CTCGAG**ATTGGATTGGATTGCCAAAT |
|  | PBtaI2R | CGC**GGATCC**CTTGACGGTGGAATCCAGTT |
| ***btaI*3** | PBtaI3F | CCG**CTCGAG**GCGATGGAGAAGCTCAACAC |
|  | PBtaI3R | CG**GGATCC**TGCGGTTTCGAAGGCTGT |
