# Supplementary material for: The Complex Quorum Sensing Circuitry of Burkholderia thailandensis Is Both Hierarchically and Homeostatically Organized
Source: mBio. 2017 Dec 5;8(6):e01861-17. doi: 10.1128/mBio.01861-17 (PMC5717390; doi:10.1128/mBio.01861-17)
Supplement: TABLE S4 [file mbo006173620st4.docx]

**Table S4. Primers used for qRT-PCR.**

| **Genes** | **Oligonucleotides** | **Sequences (5’ to 3’)** |
| --- | --- | --- |
| ***ndh*** | SLG_qRT-PCR_ndh_F | ACCAGGGCGAATTGATCTC |
|  | SLG_qRT-PCR_ndh_R | GATGACGAGCGTGTCGTATT |
| ***btaR*1** | SLG_qRT-PCR_btaR1_F | AGCTCGAACATGATCGTCTG |
|  | SLG_qRT-PCR_btaR1_R | TGAAGCGTCAGATGGTTGAT |
| ***btaR*2** | SLG_qRT-PCR_btaR2_F | GAGAAATTCCGCAACGAGAG |
|  | SLG_qRT-PCR_btaR2_R | GCCGTCCACTTCAACACAT |
| ***btaR*3** | SLG_qRT-PCR_btaR3_F | CGACTACTTCACCATCGATCC |
|  | SLG_qRT-PCR_btaR3_R | GCTGATGCCGTTGTCGAG |
